# Combining the Specific Anti-MUC1 Antibody TAB004 and Lip-MSA-IL-2 Limits Pancreatic Cancer Progression in Immune Competent Murine Models of Pancreatic Ductal Adenocarcinoma

**DOI:** 10.3389/fonc.2019.00330

**Published:** 2019-04-30

**Authors:** Didier Dréau, Laura Jeffords Moore, Mike Wu, Lopa Das Roy, Lloye Dillion, Travis Porter, Rahul Puri, Noor Momin, K. Dane Wittrup, Pinku Mukherjee

**Affiliations:** ^1^Department of Biological Sciences, UNC Charlotte, Charlotte, NC, United States; ^2^OncoTab Inc., Charlotte, NC, United States; ^3^Koch Institute for Integrative Cancer Research, Massachusetts Institute of Technology, Cambridge, MA, United States

**Keywords:** ADCC, Antibody, IL-2, Immunocompetent, Immunotherapy, MUC1, PDA, TAB004

## Abstract

Immunotherapy regimens have shown success in subsets of cancer patients; however, their efficacy against pancreatic ductal adenocarcinoma (PDA) remain unclear. Previously, we demonstrated the potential of TAB004, a monoclonal antibody targeting the unique tumor-associated form of MUC1 (tMUC1) in the early detection of PDA. In this study, we evaluated the therapeutic benefit of combining the TAB004 antibody with Liposomal-MSA-IL-2 in immune competent and human MUC1 transgenic (MUC1.Tg) mouse models of PDA and investigated the associated immune responses. Treatment with TAB004 + Lip-MSA-IL-2 resulted in significantly improved survival and slower tumor growth compared to controls in MUC1.Tg mice bearing an orthotopic PDA.MUC1 tumor. Similarly, in the spontaneous model of PDA that expresses human MUC1, the combination treatment stalled the progression of pancreatic intraepithelial pre-neoplastic (PanIN) lesion to adenocarcinoma. Treatment with the combination elicited a robust systemic and tumor-specific immune response with (a) increased percentages of systemic and tumor infiltrated CD45+CD11b+ cells, (b) increased levels of myeloperoxidase (MPO), (c) increased antibody-dependent cellular cytotoxicity/phagocytosis (ADCC/ADCP), (d) decreased percentage of immune regulatory cells (CD8+CD69+ cells), and (e) reduced circulating levels of immunosuppressive tMUC1. We report that treatment with a novel antibody against tMUC1 in combination with a unique formulation of IL-2 can improve survival and lead to stable disease in appropriate models of PDA by reducing tumor-induced immune regulation and promoting recruitment of CD45+CD11b+ cells, thereby enhancing ADCC/ADCP.

## Introduction

Pancreatic ductal adenocarcinoma (PDA) has the poorest prognosis of all malignancies with more than 260,000 deaths annually worldwide, a 5% 5-year survival rate, a mean life expectancy of <6 months, and a high degree of resistance to standard therapy (1–4). Radiotherapy and chemotherapy remain largely ineffective. While surgery is an option, only 20% of PDA patients have resectable tumors at the time of diagnosis and the recurrence rate remains high in these patients. In addition to surgery, PDA is treated with adjuvant therapies including gemcitabine to reduce the incidence of local recurrences and distant metastases (3, 5). Combination treatments such as rosiglitazone and gemcitabine, FOLFIRINOX (5-FU, leucovorin, irinotecan, and oxaliplatin), monoclonal antibody (mAb), and 5-fluorouracil, or gemcitabine and nab-paclitaxel have been shown to significantly reduce tumor progression and metastases and significantly extend overall patient survival (1, 6–8). While those treatments led to some improvements and extended overall survival in small subsets of patients (from 6–7 months to ~25 months) (8, 9), improved approaches to treat patients with pancreatic cancer remain urgent (3, 10).

Cancer immunotherapies that target tumor associated antigens present attractive alternatives as these approaches are expected to cause fewer side effects while preventing metastasis and recurrence better than standard therapies. Antibody-based immunotherapy for cancer was established within the past 15 years, and is now one of the most successful strategies for treating patients with hematological and solid tumors (11). The fundamental basis of antibody-based therapy of tumors relies on the presence of cell surface antigens that are overexpressed, mutated or selectively expressed compared with normal tissues (11). A key challenge has been to identify antigens that are suitable for antibody-based therapeutics. There are approximately 460 active clinical trials with 38 antibody-based drugs and several new products under development. Some examples of FDA approved antibodies for solid tumors include Herceptin®, Avastin®, Erbitux®, Vectibix®, and Ipilimumab®. However, none is approved for pancreatic cancer.

There is clinical evidence for mAb driven T cell immunity. For instance, the therapeutic effect of rituximab was augmented by eliciting a T cell response (12). Further, administration of cetuximab triggered expansion of EGFR-specific T cells (13); and trastuzumab elicited a Her-2/neu-specific cellular response (14). Since interleukin-2 (IL-2) through enhancement of NK ADCC greatly improved the therapeutic efficacy of mAbs (15, 16), trials with trastuzumab and rituximab in combination with IL-2 were conducted. The results were disappointing, with little to no objective clinical response observed with the combination (17, 18). This is most likely because IL-2 in its native form is short lived *in vivo* and increasing the dose is toxic. Indeed, combining antibodies with a form of IL-2 with extended circulation provided surprisingly robust control of B16 melanoma tumor growth, in the absence of any marked toxicity (19). Administration of IL-2, which supports the survival and function of tumor-reactive T cells (20), has been shown to benefit some patients with melanoma (21). However, the vascular leak syndrome associated with the high-dose IL-2 treatment regimen has limited its use in tumor immunotherapy (21). More recently, Lip-MSA-IL-2, a formulation stabilizing IL-2, was associated with the generation of an immune response that prevented melanoma progression in a murine model (22).

Mucin-1 (i.e., MUC1, CD227) is a membrane-tethered mucin overexpressed and aberrantly glycosylated in many epithelial malignancies, including >90% of human PDA (23–29). The hypo-glycosylated MUC1 expressed on malignant cells renders normally cryptic MUC1 epitopes open to detection and is hereto forth referred to as tMUC1. MUC1 has long been an interesting target molecule for immunotherapy development, given its highly increased cell surface expression and altered glycosylation in tumors [reviewed in (30)]. Many antibodies have been developed that recognize epitopes of those tumor-associated hypo-glycosylated MUC1 regions, including PankoMab, Pemtumomab (also known as HMFG1) and TAB004 (26, 27, 31–33). TAB004 (patent #8,518,405, and 9845362 B2) was initially developed using pancreatic tumors expressing the altered form of MUC1 (34). TAB004 targets the epitope area (AA950-958) which is only accessible for antigenic detection in cells expressing the hypo-glycosylated form of MUC1 (35–38). In contrast to most other MUC1 antibodies, TAB004 distinguishes between normal and tumor-associated forms of MUC1 by relying solely on the expression of hypo-glycosylated MUC1. Further, TAB004 was effective in identifying primary PDA and pancreatic cancer stem cells in PDA patients, while sparing recognition of normal tissue (27, 39).

Previously, we have demonstrated the effectiveness of MUC1-directed tumor vaccines in colorectal, pancreatic, and breast cancer models (38, 40, 41); however, immunosuppression within the tumor microenvironment hindered the effectiveness of the vaccine (41). We have recently shown that an anti-MUC1 antibody can be used as a therapeutic antibody when conjugated to the immune modulating agent CpG ODN via enhanced NK cell anti-tumor activity against PDA tumors (42).

Here we sought for the first time to determine whether the combination of TAB004 and stabilized Lip-MSA-IL-2 elicits an immune response and confers a survival benefit in orthotopic and spontaneous immunocompetent murine models of PDA. Our results indicate that, with minimal toxicity, the combination of TAB004 + Lip-MSA-IL-2 was associated with improved survival in the orthotopic murine model of PDA, as well as a lower cancer burden in the PDA.MUC1 mouse spontaneous model of PDA.

## Materials and Methods

### TAB004 Antibody and Lip-MSA-IL-2

The antibody TAB004 has been described earlier (27, 43) (OncoTab Inc., Charlotte NC). The stabilized Lip-MSA-IL-2 has been described (22) and was provided by Dr. Wittrup (Massachusetts Institute of Technology, Cambridge, MA). The optimal dose of TAB004 (500 μg/mouse/injection i.e., 25 mg/kg/dose) was determined in preliminary experiments using doses ranging from 62.5 to 1,000 μg/mouse/injection. The dose of Lip-MSA-IL-2 used (25 μg/mouse/injection, i.e., 1.25 mg/kg/dose) was derived from previous experiments (22).

### KCM and KCM-LUC+ PDA Cells

KCM cells were generated from spontaneous PDA tumors from PDA.MUC1 triple transgenic mice (LSL-Kras^G12D^ X ^P48^Cre X human MUC1.Tg mice) (44) and, therefore, express human MUC1 (43, 45). The KCM-Luc cell line was generated by retroviral transduction of KCM cells with the MSCV Luciferase PGK-Hygro plasmid (Addgene plasmid # 18782, a generous gift from Scott Lowe, Memorial Sloan Kettering Cancer Center, New York, NY) (46). Both KCM and KCM-Luc+ cells were cultured and expanded in DMEM (Gibco, Waltham, MA) supplemented with 10% fetal bovine serum (FBS, Gibco), glutamine, penicillin, and streptomycin (Cellgro, Corning, Manassas, VA).

### Spontaneous Mouse Model of PDA

Triple transgenic mice (i.e., PDA.MUC1 also designated KCM mice) express human MUC1 as a self-molecule and is the first model of invasive pancreatic cancer that expresses human MUC1 (44). Indeed, KCM mice develop ductal lesions with complete penetrance (100%), very similar to all three stages of human pancreatic intraepithelial neoplasia (PanIN) lesions (PanIN-1A, PanIN-1B, PanIN-2, and PanIN-3) and progress to adenocarcinoma and lung metastasis. As early as 6–16 weeks of age, mice develop PanINs of different stages including PanIN-IA, PanIN-IB, and PanIN-2. By 20–26 weeks of age, early PanIN lesions progress to PanIN-3 and carcinoma *in-situ* and by 30–36 weeks, invasive adenocarcinoma and metastasis are observed. As in human PDA, tumor cells express high levels of tMUC1 (44) that were detectable using the TAB004 antibody (27). KCM mice are characterized by (1) tumors arising spontaneously in the pancreatic ductal epithelial cells due to the KRAS^G12D^ mutation; (2) the normal human MUC1 transformed to tMUC1 with disease progression just as observed in the human disease; (3) tumors arising in fully immune competent host; and (4) tolerance to MUC1 immunization as MUC1 is expressed as a self-molecule driven by its own regulatory sequence (47).

**Figure 1:**
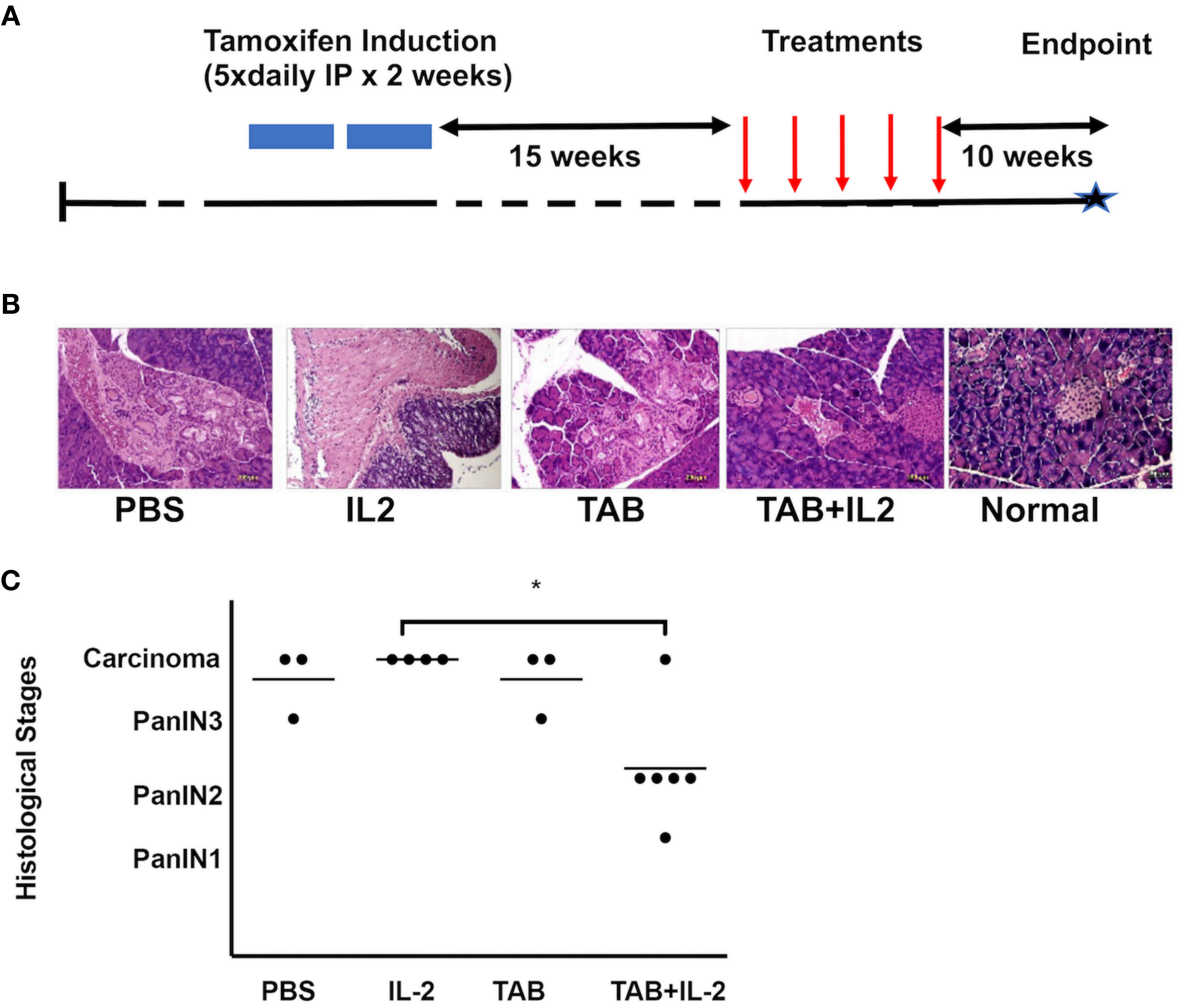
TAB004 + Lip-MSA-IL-2 treatment significantly slowed tumor progression in a spontaneous pancreatic cancer mouse model. KrasG12Dmut; P-48Cre; MUC1.TG (KCM) triple-transgenic 8–16 week-old mice were induced with Tamoxifen (75 mg/kg, IP, for 5 consecutive days for 2 weeks). Following tamoxifen induction, all KCM mice develop pancreatic cancer lesions around 30–40 week of age (41, 44). **(A)** Tamoxifen-induced 23–31 week-old mice were administered once weekly either PBS (*n* = 3), Lip-MSA-IL-2 (*n* = 4), TAB004 (*n* = 3), or the combination TAB004 + Lip-MSA-IL-2 (*n* = 6). Ten weeks later, pancreata were collected and processed for histology. Pancreas slides (5–6 μm thick) from each mouse were stained using hematoxylin and eosin and the presence of Pan lesions and/or carcinoma blindly assessed and recorded. **(B)** Representative micro-photographs of H&E stained pancreas sections from normal and tamoxifen-induced KCM mice treated with PBS, Lip-MSA-IL-2, TAB004, and TAB004 + Lip-MSA-IL-2. Note the presence of carcinoma in all pancreases except those of normal and TAB004 + Lip-MSA-IL-2 treated mice. **(C)** Each pancreas was evaluated using the pancreatic cancer histological stages, i.e., PanIN1, PanIN2, PanIN3, and carcinoma. ^*^p < 0.05. TAB, TAB004; IL2, Lip-MSA-IL-2; IP, intraperitoneal.

Following an Institutional Animal Care and Use Committee (IACUC) approved protocol, KCM triple transgenic mice were primed to activate the KRAS mutation through a CRE tamoxifen sensitive cassette (41, 44) during week 12–13 of age. All KCM mice treated with tamoxifen (20 mg/ml/mouse 5 day/week for 2 weeks) develop PanIN lesions by 20–23 week of age. At that age, animals (*n* = 18) were randomized and treated with either PBS (vehicle, *n* = 4), TAB004 alone (*n* = 4), Lip-MSA-IL-2 (*n* = 4) alone or the combination of TAB004 + Lip-MSA-IL-2 (*n* = 6; for treatment schedule and dose, see Figure 1A). Two animals died or were removed from the study per IACUC guidelines: one in the PBS group and one in the TAB004 alone group, respectively. Animals were treated for 5 weeks and monitored daily for health concerns. Body weight was recorded weekly and all mice were euthanized at 36–40 week of age. At euthanasia, pancreata were collected free of fat and surrounding tissue, fixed in 10% buffered formalin and embedded in paraffin. Pancreas sections (4–6 μm) were stained with hematoxylin and eosin (H&E), and the presence of PanIN lesions and/or adenocarcinoma was determined following a microscopic assessment of 5 sections per pancreas. For each animal, pancreatic lesions were counted and scored in 10 microscope fields (100x) and for each pancreas, the most advanced stage was reported (41, 44).

### Orthotopic Mouse Model of PDA

**Figure 2:**
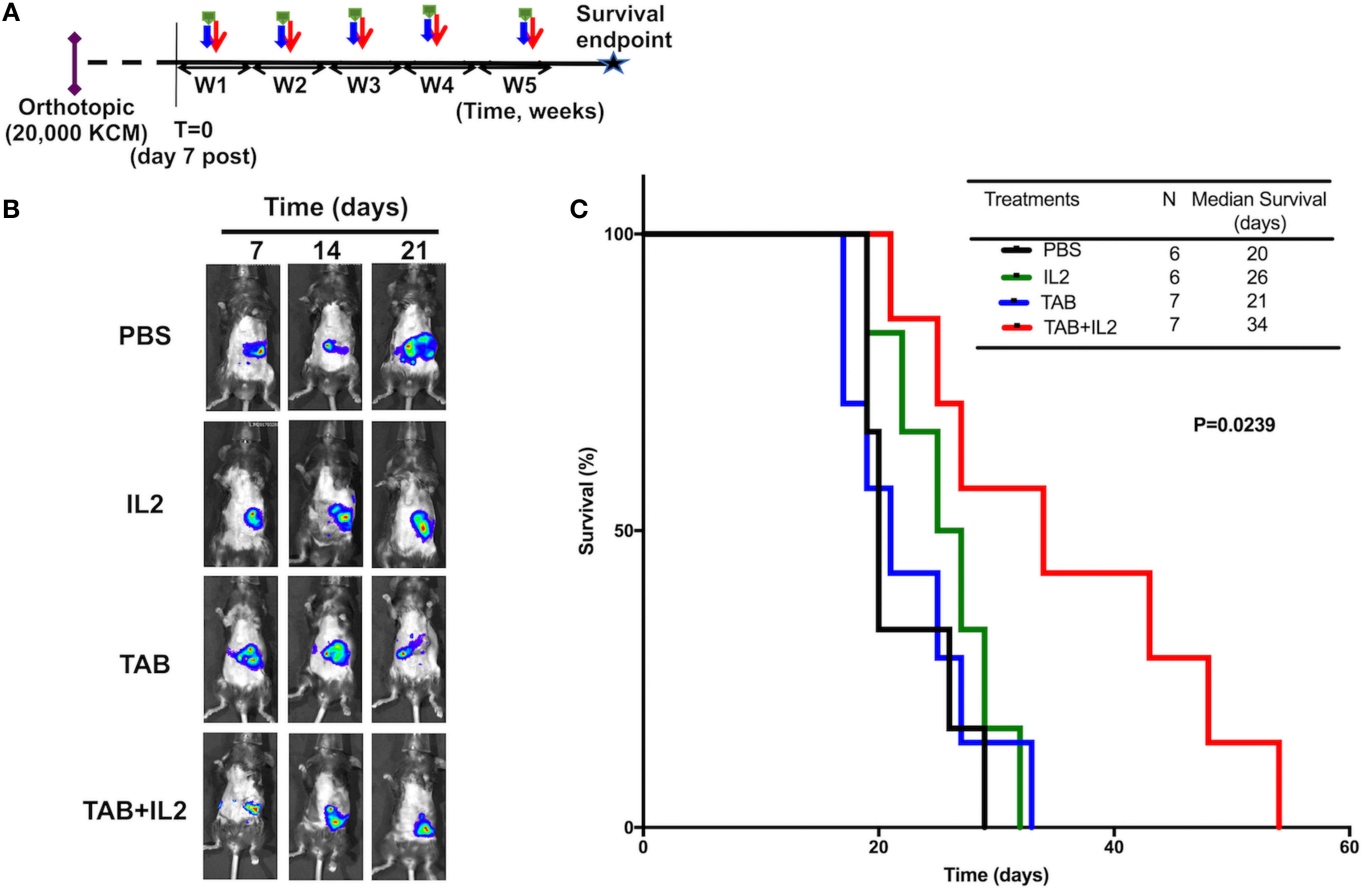
TAB004 + Lip-MSA-IL-2 treatment significantly slowed tumor progression in the KCM orthotopic pancreatic cancer mouse model. MUC1.Tg mice were implanted orthotopically with KCM-Luc+ pancreatic cancer cells (20,000 cells) and treated [see schedule **(A)**; details in Materials and Methods section]. Mice implanted orthotopically with KCM-Luc+ cells were treated with either PBS, Lip-MSA-IL-2, TAB004, or the combination TAB004 + Lip-MSA-IL-2. The tumor growth was monitored *in vivo* through luciferase bioluminescence imaging. **(B)** Representative IVIS bioluminescent images of mice treated with PBS, Lip-MSA-IL-2, TAB004, or the combination TAB004 + Lip-MSA-IL-2 at days 7, 14, and 21 post-tumor implantation, respectively (One mouse per treatment group shown from baseline i.e., day 7–day 21). **(C)** Kaplan Meier survival curves of mice (*n* ≥ 6 mice/group) implanted orthotopically with KCM-luc+ pancreatic tumor cells (20,000 cells) treated with PBS, TAB004, Lip-MSA-IL-2, or the combination TAB004 + Lip-MSA-IL-2 (Log-rank test, *p* = 0.0239). TAB, TAB004; IL2, Lip-MSA-IL-2; W, week.

**Figure 3:**
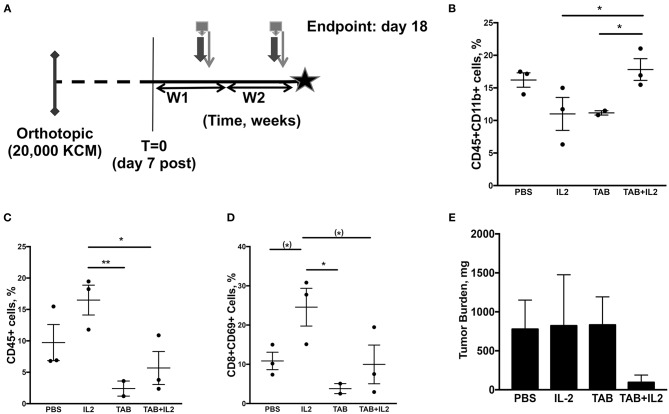
Tumor-infiltrating immune cell populations, particularly macrophages, were altered in mice treated with the combination TAB004 + Lip-MSA-IL-2. MUC1.Tg mice were implanted orthotopically with KCM-Luc+ pancreatic cancer cells (20,000 cells) and treated with either PBS, Lip-MSA-IL-2, TAB004, or the combination TAB004 + Lip-MSA-IL-2 [see schedule **(A)**; details in Materials and Methods section] for 18 days. On day 18, tumor cell suspensions were obtained and the presence (%) of CD45+ immune cells **(C)** CD45+CD11b+ cells **(B)**, and of activated effector T cells (CD45+CD8+CD69+) **(D)** was determined. Additionally, tumor burden **(E)** was recorded [*n* = 3 per treatment group except for TILs evaluations **(B–D)** in TAB group, *n* = 2]. For **(B–E)**, data are presented as mean ± SEM. (^*^)*p* < 0.08, ^*^p < 0.05, ^**^p < 0.01. TAB, TAB004; IL2, Lip-MSA-IL-2; W, week.

Surgeries were performed in a sterile environment under the supervision of the UNCC attending veterinarian and IACUC approved protocols. The resident veterinarian (Dr. Williams, DVM) orthotopically injected 20,000 KCM-Luc+ cells (~50 μl) in the pancreas of both male and female human MUC1.Tg mice (originally received from Dr. Gendler, Mayo Clinic, Arizona and bred in-house at UNC Charlotte) (47). When implanted orthotopically in those mice, KCM cells generate tumors (45). The optimal number of cells to be injected was determined in a preliminary experiment using 10,000–200,000 KCM-Luc+ cells. Following surgical healing (i.e., on day 7 post-surgery), the presence of KCM-Luc+ tumors in mice was assessed using an IVIS system (Perkin Elmer). Treatment started on day 8 post-surgery and included four groups: vehicle (PBS), TAB004, Lip-MSA-IL-2, or the combination TAB004 + Lip-MSA-IL-2. Treatments were administered IP once weekly and tumor progression was monitored by chemiluminescence through weekly IVIS imaging. Detailed schedules and dose are provided in Figures 2A, 3A for survival and mechanistic studies, respectively.

Mice were weighed weekly and monitored for activity level and adverse health events daily. Loss of more than 20 percent of body weight led to the euthanasia of the animal at which time tumor and serum were collected. For the survival studies, animals (*n* = 6–7 per treatment group) were treated for up to 5 weeks. For the mechanistic study, animals (*n* = 3 per treatment group) were euthanized at day 18 based on preliminary studies in which control animals (vehicle-injected group) had to be removed from the study by day 19–22 due to high tumor burden and morbidity. No TILs were available for one animal from the TAB004 group. For all animals, tumors and surrounding pancreas, spleen, and blood were collected. For the mechanistic studies, cells from spleen, tumor, and blood were used for ADCC/ADCP assays, flow-cytometry analyses of tumor infiltrating lymphocytes, and of systemic immune cells. In addition, sera were assayed for cytokines, myeloperoxidase (MPO) and the presence of tMUC1. Collected whole blood was used to determine cell blood counts (CBC; IDEXX, Columbia, MO).

### Flow Cytometry Analyses of Blood and Intra-Tumoral Immune Cells

Blood, spleen and tumors, obtained on day 18 post-tumor implantation from animals orthotopically implanted with KCM-Luc+ cells and treated as detailed above, were assessed by flow cytometry for specific subsets of lymphocytes. Tumor cell suspensions were obtained following mechanical disruption of the tumor mass and filtration through 70 μm strainers (BD Biosciences San Jose, CA). Non-necrotic areas were used to generate cell suspensions, and cell suspensions were further treated 15 min with DNAse1 (10 μg/ml). Tumor cell suspensions were washed, counted and resuspended in PBS (1 × 10^7^ cells/ml). For each sample, 10^6^ cells were stained for CD45 (anti-CD45-APC), CD4 (anti-CD4-FITC), CD8 (anti-CD8-PE), CD69 (anti-CD69-PE-cy7), NK (anti-NK-1.1-PE), CD107 (anti-CD107-FITC), anti-ly6G-PE, and/or CD11b-FITC. Ly6G and CD11b have been used to identify and deplete neutrophils (48) and macrophages (49) predominantly, respectively. Of note, CD11b in particular is also expressed on the surface of other immune cells (50). Corresponding isotype controls for APC, PE, FITC, PE-Cy7 were run concurrently. All antibodies were purchased from BD-Biosciences. Additionally, cells were stained to exclude dead cells using Fixable Viability Dye (FVD, eBioscience, CA) (51). Samples were run on a Fortessa flow-cytometer (BD Biosciences) and the data analyzed using FlowJo software (BD Biosciences).

### Systemic Cytokines

Sera obtained on day 18 post-tumor implantation from animals orthotopically implanted with KCM-Luc+ cells and treated as detailed above, were assessed for the following 20 cytokines: GMCSF, IL-1α, IL-2, IL-4, IL-6, IL-10, IL-13, CXCL1, M-CSF, TNF-α, IL-1β, IL-3, IL-5, IL-9, IL-12, IL-17, MCP1, RANTES, and VEGF (Quantibody® Mouse Cytokine Array 1; Raybiotech) according to the manufacturer's recommendations. Following incubations, washing, and detection steps, fluorescent signal was detected using a Tecan LS300 Series Scanner (Tecan, San Jose, CA).

### Systemic Myeloperoxidase

The concentrations of MPO, a phagocyte hemoprotein (that primarily mediates host defense reactions) abundantly expressed in neutrophils and moderately expressed in macrophages and secreted during their activation (52, 53), was determined by ELISA (Boster Biological Tech, CA). MPO levels were determined in sera obtained on day 18 post-tumor implantation. MPO concentrations expressed in pg/ml of sera were derived from a standard curve that was run along with the samples tested.

### Blood Cell Counts

White blood cells counts, RBC counts and features were determined by IDEXX Bioresearch (Columbia, MO) on whole blood, obtained on day 18 post-tumor implantation from animals orthotopically implanted with KCM-Luc+ cells and treated as detailed above.

### Antibody-Dependent Cell Cytotoxicity (ADCC)/Antibody-Dependent Cell Phagocytosis (ADCP)

ADCC/ADCP was evaluated by flow cytometry as detailed previously (54). Briefly, target cells (KCM) were labeled with carboxyfluorescein succinimidyl ester (CFSE, BioLegend 488 nm) dye for ~5 min, seeded in 24 well tissue culture plates and incubated overnight (37°C, 5% CO_2_, humidity >80%) (55, 56). Splenocytes were added to target cells at a 1:5 tumor:splenocyte ratio in the presence of TAB004 (0.1–1.0 μg/ml). Maximum lysis was obtained following incubation with saponin 0.1%. After a 24 h incubation, cells were harvested and stained with the viability dye Vital fluorophore (Fixable Viability Dye ~360–405 nm, BioLegend) (51). Cells were then run on a Fortessa flow cytometer (BD Biosciences), and gating on CFSE+ cells, percent of dead KCM cells were determined.

### tMUC1 Concentrations

Concentrations of tMUC1 in the serum obtained on day 18 post-tumor implantation from animals orthotopically implanted with KCM-Luc+ cells and treated as detailed above, were determined by ELISA as previously described (27, 43, 57).

### Statistical Analyses

Data are presented as mean ± SEM. Survival differences between treatments were represented using Kaplan-Meier and tested using Log-rank tests. Other parameters measured (e.g., % stained cells, tumor weight, % of lysis, number and type of pancreatic lesions, MPO, and MUC1 concentrations) were assessed for normality using the Shapiro-Wilk normality test. For parameters with normal distribution, differences between treatment groups were tested using ANOVA and *post-hoc* tests. Correlations between tumor weight and parameters measured was assessed by Pearson r correlations. All analyses were completed using Prism 7 (GraphPad software Inc.). *A priori p* < 0.05 was defined as significant.

## Results

### Treatment With TAB004 + Lip-MSA-IL-2 Limited Pancreatic Cancer Progression in the KCM Triple Transgenic Mice That Develop Spontaneous PDA

The effects of TAB004 with or without Lip-MSA-IL-2 were assessed in the KCM mice, a model of human MUC1-expressing spontaneous PDA. KCM mice carry the human MUC1 transgene (driven by its own promoter) and the KRAS^G12D^ transgene (driven by tamoxifen-inducible P48 promoter) (41, 44). When KCM mice are injected with tamoxifen for 2 weeks starting at 11 weeks of age, all (100%) mice develop early stage PanIN lesions by 20–24 weeks of age (~12 weeks post tamoxifen) (41, 44).

KCM mice were treated with vehicle (PBS: *n* = 3), TAB004 (500 μgs/mouse. *n* = 3), Lip-MSA-IL-2 (25 μgs/mouse. *n* = 4), or TAB004 + Lip-MSA-IL-2 (*n* = 6) for 5 weeks starting 15 weeks post-tamoxifen treatment (or 26 weeks of age) (Figure 1A). At 40 weeks of age, pancreata were harvested and processed for histology. Tumor grade was determined following hematoxylin and eosin (H&E) staining. Representative microphotographs of H&E stained pancreas sections from each treatment group compared to normal pancreas highlight the presence of tumor lesions (Figure 1B). Results show that in 5 out of 6 mice treated with the combination TAB004 + Lip-MSA-IL-2, the PanIN lesions did not progress beyond the PanIN2 grade. One mouse in the combination TAB004 + Lip-MSA-IL-2 group progressed to adenocarcinoma. In sharp contrast, all 4 mice treated with Lip-MSA-IL-2 alone progressed from PanIN lesions to adenocarcinoma. Similarly, 2 out of 3 mice in the PBS group and in the TAB004 group progressed to adenocarcinoma while one mouse from each group progressed to high-grade PanIN3/CIS grade (Figure 1C). Of note, we did not observe any adverse effect of the treatment on the health of the KCM mice.

### Treatment With TAB004 and Lip-MSA-IL-2 Also Significantly Improved Survival of MUC1.Tg Mice Bearing Orthotopic KCM Tumors

MUC1.Tg mice bearing orthotopic KCM.Luc+ pancreatic tumors were treated with either vehicle (PBS), TAB004, or Lip-MSA-IL-2 alone or the combination of TAB004 + Lip-MSA-IL-2 weekly for up to 5 weeks (Figure 2A, *n* ≥ 6 mice per treatment group). The *in vivo* growth of KCM cells expressing luciferase was monitored post-luciferin injection as shown in representative IVIS images of orthotopic tumor (one mouse per treatment over time from day 7 to day 21, Figure 2B). By day 21, while tumors grew in all mice, reduced bioluminescence indicative of smaller tumors was detected in treated mice groups, suggesting that those tumors grew more slowly compared to tumors in mice treated with PBS (Figure 2B). More importantly, TAB004 + Lip-MSA-IL-2 treatment was associated with a significantly improved survival (*p* = 0.02, Log rank test, Figure 2C) compared to mice that received any other treatments. Notably, TAB004 alone or Lip-MSA-IL-2 alone did not improve mouse survival.

No significant toxicity was associated with the treatments except for mild to severe skin dermatitis observed in a third of the mice treated with Lip-MSA-IL-2 alone and half of the mice treated with the TAB004 + Lip-MSA-IL-2 combination. Complete blood cell count (WBC and RBC) analyses were conducted on whole blood. No difference was observed in RBC measured parameters (Supplemental Figure 1S) and WBC populations (Supplemental Figure 2S) when comparing tumor bearing and treated mice with control non-tumor bearing MUC1.Tg mice.

### Treatment With TAB004 + Lip-MSA-IL-2 Was Associated With Increases in CD45+CD11b+ Cells and Decreases in Both CD45+ Lymphocytes and CD8+CD69+ T Cells Within the Orthotopic KCM Tumors

In another set of experiments, tumors were collected 18 days post tumor challenge and 2 weeks post treatment (Figure 3A) to assess the treatment induced immune responses (*n* = 3 mice per treatment group). Changes in specific immune cell populations including macrophages, neutrophils, NK cells, lymphocytes, and lymphocyte subsets are associated with effective immunotherapy (58, 59). In particular, IL-2 treatment is associated with increases in neutrophils and activated NK (NK1.1+CD107+) cells (19). Moreover, tumor infiltration by specific subsets of macrophages is also associated with improved pre-clinical responses (60, 61). Therefore, we assessed immune cell subpopulations in spleen, blood and tumors from MUC1.Tg mice orthotopically implanted with KCM tumor cells, treated as detailed in Figure 3A. In the spleen, there was no significant difference in the populations of CD45+, CD8+, CD4+, CD4+CD69+, CD8+CD69+, NK1.1+, NK1.1+CD107+, CD11b+, or Ly6G+ as determined by flow cytometry (data not shown).

In the tumors, a significant increase in the percent of tumor-associated CD45+CD11b+ cells was observed in the tumors of mice treated with the combination of TAB004 + Lip-MSA-IL-2 compared to tumors from mice in all other treatments (*p* < 0.01; Figure 3B). Interestingly, in mice treated with the combination TAB004 + Lip-MSA-IL-2 or TAB004 alone, we observed a significant decrease in the percent of tumor-associated CD45+ lymphocytes and of CD8+CD69+ T lymphocytes compared to tumors from mice treated with PBS or Lip-MSA-IL-2 alone (*P* < 0.01; Figures 3C,D). A subset of CD8+CD69+ cells has been identified as activated CD8+ regulatory cells previously (62). Although tumor infiltrating CD45+ cells were lower in the mice treated with the TAB004 + Lip-MSA-IL-2 combination, the CD45+CD11b+ cell population remained high. There was no significant change in any other subpopulation including Tregs, CD4+CD69+, CD8+, NK1.1+, or activated NK cell populations (data not shown). The tumor weight was also assessed at day 18 and was highly variable. Nevertheless, the orthotopic tumor burden in the mice treated with the combination TAB004 + Lip-MSA-IL-2 tended to be smaller compared to the tumor burden observed the other treatment groups (ns, Figure 3E).

Of note, the number of monocytes per μl of blood was significantly decreased in mice treated with the combination of TAB004 + Lip-MSA-IL-2 compared to mice treated with Lip-MSA-IL-2 alone (Supplemental Figure 3AS). Additionally, the number of CD45+CD11b+ cells per gram of tumor was higher in tumors isolated from animals treated with Lip-MSA-IL-2 alone compared tumors collected from mice treated with PBS or TAB004 (Supplemental Figure 3BS).

Together, the survival and mechanistic experiments conducted in the orthotopic KCM tumor model highlight that the tumor progression is slower and the tumor burden is lower in the mice treated with the combination TAB004 + Lip-MSA-IL-2 compared to mice treated with PBS, TAB004 or Lip-MSA-IL-2 alone. Furthermore, a significant survival benefit was observed in the mice treated with the combination TAB004 + Lip-MSA-IL-2 (see Figure 2C above).

### The Combination TAB004 + Lip-MSA-IL-2 Treatment Markedly Increased the Myeloperoxidase Present in the Serum of MUC1.Tg Mice Bearing Orthotopic KCM Tumors

**Figure 4:**
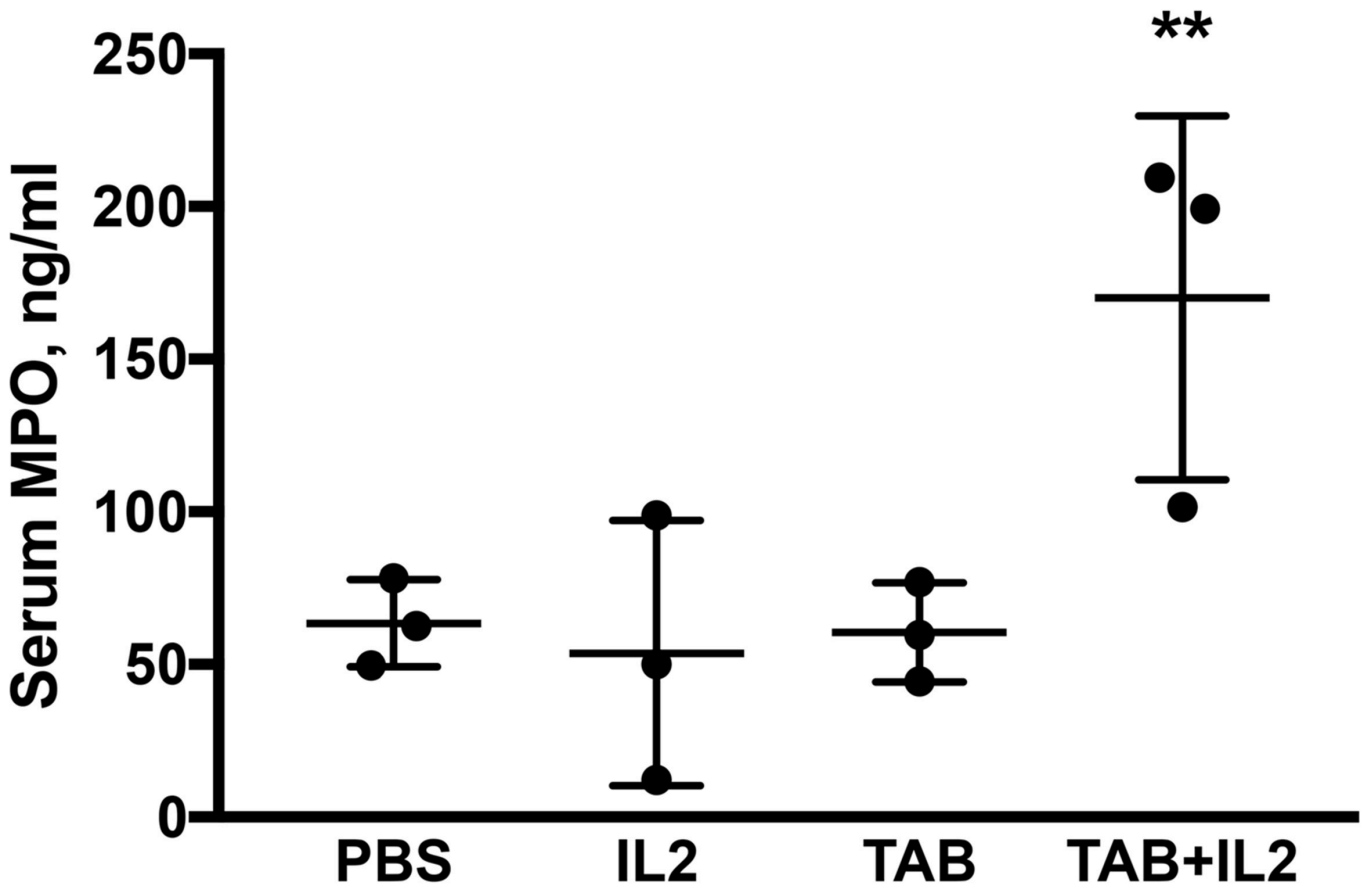
Blood myeloperoxidase (MPO) concentrations were significantly higher in KCM-tumor bearing mice following TAB004 + Lip-MSA-IL-2 treatment. Sera were assessed by ELISA for the presence of MPO and the MPO concentration expressed in pg/ml (*n* = 3 per treatment group). Data are presented as mean ± SEM. ^**^p < 0.01. TAB, TAB004; IL2, Lip-MSA-IL-2.

Multiple immune cells, especially neutrophils and macrophages, have a MPO activity (52, 53). MPO is produced during degranulation of neutrophils and macrophages and produces hypochlorous acid that is generally associated with cellular cytotoxicity. MPO concentrations were determined using ELISA in sera. Significantly higher MPO concentrations were detected in the sera of KCM tumor bearing mice treated with the combination of TAB004 + Lip-MSA-IL-2 than in the sera of the mice that were administered the other treatments (*P* < 0.001; Figure 4).

### The Combination TAB004 + Lip-MSA-IL-2 Treatment Led to Significant Increases in Circulating Levels of CXCL1 and IL-5 as Well as Decreased IL-6 in the Sera of KCM Tumor Bearing MUC1.Tg Mice

**Figure 5:**
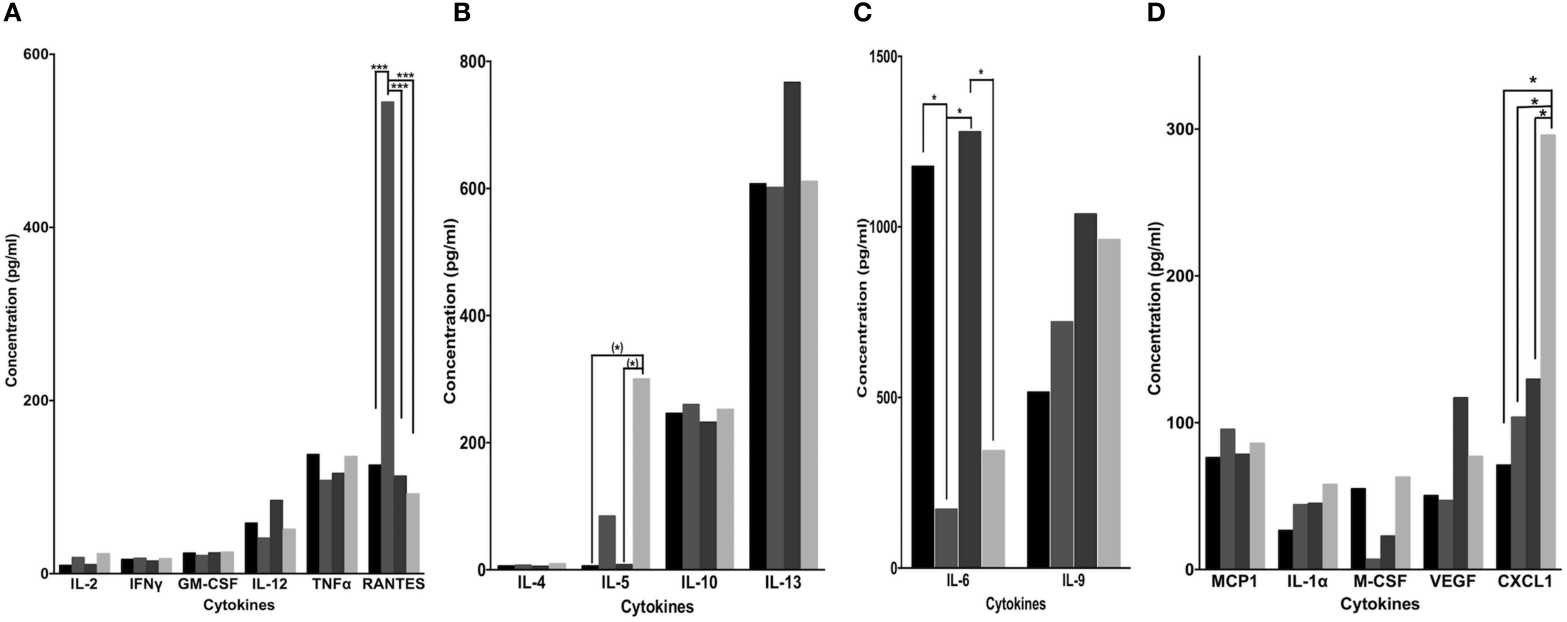
Blood cytokine concentrations following PBS, TAB004, Lip-MSA-IL-2, and TAB004 + Lip-MSA-IL-2 treatments. The presence of multiple cytokines in sera (**A–D**, pg/ml) collected from mice implanted with 20,000 KCM-luc cells and treated with PBS (black), TAB004 (dark gray), Lip-MSA-IL-2 (darker gray), and TAB004 + Lip-MSA-IL-2 (light gray) were determined using multiplex quantitative cytokine arrays (*n* = 3 per treatment group). (^*^)*p* < 0.08; ^*^p < 0.05; ^***^p < 0.001. [Values and variation (Average ± SEM) are provided in the Supplemental Table 1S].

Along with changes in immune cell infiltration, successful immunotherapies are associated with changes in multiple cytokines (63, 64). We assessed the concentrations of 20 Th1, Th2, Th17, and/or macrophage-related cytokines in sera collected 18 days post tumor challenge and 2 weeks post treatment (Figure 3A). Mice treated with the combination TAB004 + Lip-MSA-IL-2 showed a significant increase in levels of IL5 (Figure 5B) and CXCL1 (Figure 5D) compared to all other treatment groups. Following Lip MSA IL2 treatment, serum concentrations of RANTES were highly variable (Figure 5A, *p* < 0.05; Shapiro–Wilk normality test). Also noteworthy is the decrease in the serum levels of IL-6 in mice treated with Lip-MSA-IL-2 and the combination TAB004 + Lip-MSA-IL-2 compared to PBS and TAB004 treated mice (*p* < 0.05; Figure 5C).

### Treatment With TAB004 Antibody Markedly Decreased tMUC1 Serum Concentrations in the MUC1.Tg Mice Bearing Orthotopic KCM Tumors

**Figure 6:**
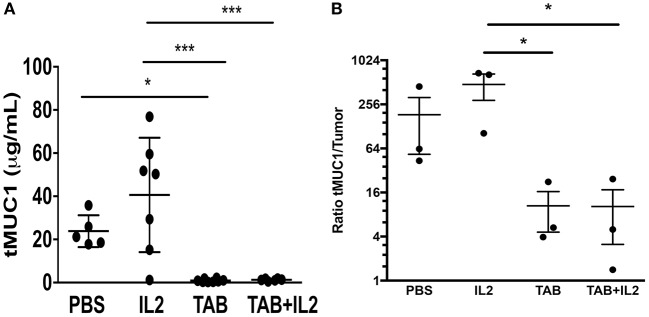
tMUC1 serum concentrations were significantly lower in TAB004 and TAB + Lip-MSA-IL-2 treated mice. tMUC1 was measured by ELISA in the serum of KCM tumor-bearing mice (**A**, *n* ≥ 5) treated with PBS, Lip-MSA-IL-2, TAB004, or the TAB004 + Lip-MSA-IL-2 combination and normalized to tumor size (**B**, *n* = 3). Data are presented as mean ± SEM. ^*^p < 0.05; *p* < 0.01; ^***^p < 0.001. TAB, TAB004; IL2, Lip-MSA-IL-2.

Because tMUC1 is associated with immune suppression (31) and increased aggressiveness of pancreatic tumors (44), we determined the concentrations of serum tMUC1 in the treated mice using a specific ELISA. As was expected, serum tMUC1 concentrations were significantly lower in TAB004 and TAB004 + Lip-MSA-IL-2 treated mice when compared to serum from mice treated with Lip-MSA-IL-2 alone or with PBS (*p* < 0.05; Figure 6A). Further, similar observations were made when the serum concentrations were normalized to tumor mass (*p* < 0.05; Figure 6B).

### Treatment With TAB004 Alone and the Combination of TAB004 + Lip-MSA-IL-2 Led to an Increased Antibody-Dependent Cell Cytotoxicity/Phagocytosis

**Figure 7:**
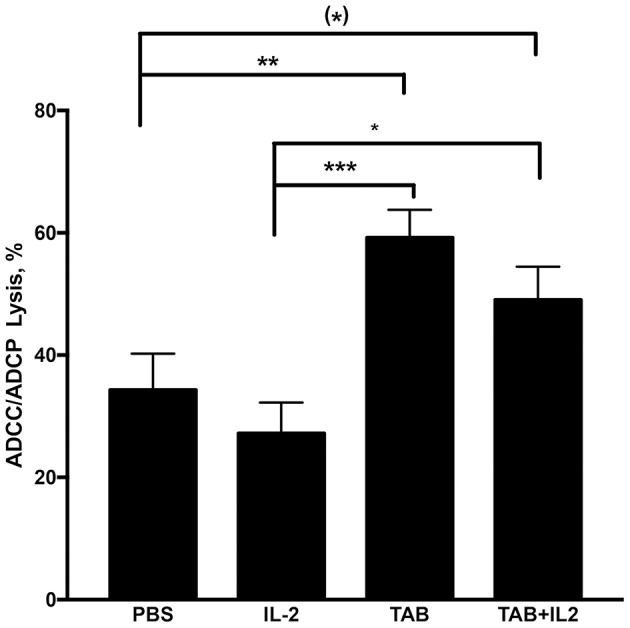
ADCC/ADCP lysis was significantly increased in mice treated with TAB004 or TAB + Lip-MSA-IL-2. Splenocytes isolated from KCM tumor-bearing mice were assayed *in vitro* for antibody-dependent cell cytotoxicity (ADCC)/antibody-dependent cell phagocytosis (ADCP) as detailed previously (54). KCM tumor cells were seeded and stained with the dye (CSFE) and incubated for 24 h with isolated splenocytes at a 1:5 tumor:splenocyte ratio in the presence of TAB004 with gating on non-CD45+ cells. The KCM cells CSFE+ dead cells (%) stained with vital fluorophore were evaluated by flow-cytometry (*n* = 3 per treatment group). Data are presented as mean ± SEM. (^*^)*p* < 0.08;^*^p < 0.05; ^**^p < 0.01; ^***^p < 0.001. TAB, TAB004; IL2, Lip-MSA-IL-2.

To further assess the cytotoxic mechanisms induced by the combination of TAB004 + Lip-MSA-IL-2, splenocytes isolated from treated KCM-tumor bearing mice (Figure 3) were assayed *in vitro* with TAB004 antibody for antibody-dependent cell cytotoxicity (ADCC)/antibody-dependent cell phagocytosis (ADCP) against KCM tumor cells as detailed previously (54). Splenocytes isolated from KCM tumor bearing mice treated with either TAB004 alone or the combination TAB004 + Lip-MSA-IL-2 had a significantly higher ADCC/ADCP response against KCM tumor cells compared to splenocytes isolated from mice treated with PBS or Lip-MSA-IL-2 alone (*p* < 0.05, Figure 7).

### Specific Immune Parameters Measured Correlated With Tumor Size and the Treatment With TAB004 Led to Lower Neutrophil/Lymphocyte Ratios

To shed light on the robustness of the association between immune variables and tumor progression in the treated mice, the immune parameters measured were correlated with tumor size regardless of treatment (Table 1). Interestingly, the immune parameters measured in the mechanistic study had significant correlations with tumor size. Specifically, a smaller tumor size was correlated with increases in CD45+CD11b+ cells present in the tumors (*r* = −0.956; *p* = 0.04), in serum MPO (*r* = −0.969; *p* = 0.03), in serum IL-5 (*r* = −0.948; *p* = 0.051), and in serum CXCL1 (*r* = −0.938; *p* = 0.06) concentrations. Additionally, a smaller tumor size was correlated with a decrease in blood neutrophil numbers (*r* = 0.969; *p* = 0.03).

**Table 1:** Pearson *r* correlation coefficients between and tumor size and immune parameters.

**Tumor size (mg) vs**.	**Pearson *r* coefficient**	**Significance**
Immune cells within tumors		
CD45+, %	0.355	n.s.
CD8+CD69+, %	0.360	n.s.
CD11b+, %	−0.956	0.04
Immune cells within blood		
WBC (Number/ul)	0.632	n.s.
Lymphocytes (Number/ul)	0.244	n.s.
Monocytes (Number/ul)	0.734	n.s.
Neutrophils (Number/ul)	0.970	0.03
Cytokines within serum		
IL-2 (pg/ml)	−0.736	n.s.
IL-4 (pg/ml)	−0.911	n.s
IL-5 (pg/ml)	−0.948	0.05
IL-9 (pg/ml)	−0.335	n.s.
CXCL1 (pg/ml)	−0.939	0.06
RANTES (pg/ml)	0.417	n.s.
Serum Muc1 (pg/ml)	0.562	n.s.
Serum MPO (pg/ml)	−0.969	0.03

**Figure 8:**
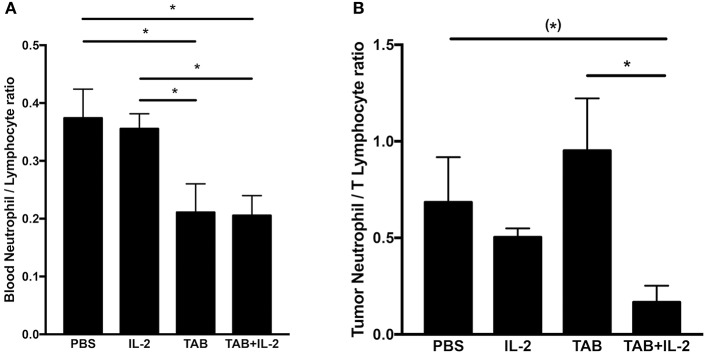
The combination TAB004 + Lip-MSA-IL-2 treatment led to lower neutrophil/lymphocyte ratio in blood **(A)** and Tumors **(B)**. Blood and tumor cells were characterized by blood count and flow-cytometry, respectively (see section Materials and Methods for details). For blood, ratios were derived using number (#) of neutrophils and lymphocytes per μl of blood. For each tumor mass, the cell suspensions obtained were stained for CD45+and ly6G+ (neutrophils) and CD45+ and either CD4+ or CD8+(T lymphocytes) evaluated by flow-cytometry and normalized to gram of tumor (*n* = 3 per treatment group). Data are presented as mean ± SEM. (^*^)*p* < 0.08;^*^p < 0.05; TAB, TAB004; IL2, Lip-MSA-IL-2.

As the blood neutrophil/lymphocyte ratio has been demonstrated to have prognostic value in monitoring tumor progression (with a lower ratio associated with improved outcomes (65, 66)), we compared the blood neutrophil/lymphocyte ratio between mouse treatment groups (Figure 8A). Treatments with either TAB004 or the combination of TAB004 + Lip-MSA-IL-2 led to significant decreases in the blood neutrophil/lymphocyte ratio compared to the blood of mice treated with PBS or Lip-MSA-IL-2 alone (*p* < 0.05, Figure 8A). Furthermore, the ratio of neutrophils (i.e., CD45+Ly6G+ cells per gram of tumor) to T lymphocytes (defined as the sum of CD45+CD4+ and CD45+CD8+ per gram of tumor) tended to be lower in the tumors of mice treated with the combination of TAB004 + Lip-MSA-IL-2 compared to tumors from mice treated with the vehicle (PBS), and was lower than the neutrophil/T lymphocyte ratio in tumors from mice treated with Lip-MSA-IL-2 (*p* < 0.05; Figure 8B).

## Discussion

The most effective pre-clinical studies are conducted in immune competent spontaneous tumor models (67). Here, using both orthotopic (KCM implanted tumor cells in the pancreas) and spontaneous PDA models, we demonstrate for the first time a significant slowdown of tumor growth and improved survival following combination treatment with TAB004 and Lip-MSA-IL-2. The beneficial therapeutic effect was associated with specific immune changes, including increases in CD45+CD11b+ cells and decreases in immune regulatory lymphocytes within the tumor mass. Treatments also led to increases in the serum concentrations of MPO and of specific cytokines as well as ADCC/ADCP activities of splenocytes. The immunocompetent orthotopic pancreatic model was informative on the effects of treatments on immune responses. Indeed, notwithstanding the number of animals per group [*n* = 3 except TAB004 group (*n* = 2)] that requires a cautious interpretation of the data, significant changes in immune parameters were observed, and most were correlated to tumor size. Of note, there was minimal toxicity associated with this treatment.

Both mouse models used here mimic the development of the human tumor, including similarities in MUC1 expression, the native immune responses against MUC1 as tumors progress, and the immune suppressive microenvironment within the developing tumor (28). In these clinically-relevant models, tumors arise in an appropriate tissue background and in a host conditioned by the physiological events of neoplastic progression and tumorigenesis and in the context of a viable immune system (67). Regardless of treatment, multiple factors likely modulate the immune responses observed here including the desmoplasia and immune evasion. For example, desmoplasia is routinely observed in pancreatic tumors and hinders chemotherapy (68, 69). Indeed, in our model, collagen accumulation was observed in all treatments (Supplemental Figure 4S) and may have affected the immunotherapy tested. Numerous mechanisms of immune evasion have been identified and reviewed elsewhere (70–72). As our tumor model is not highly immunogenic, evasion mechanisms were not investigated here and will be assessed in future studies. Moreover, both mouse models express human MUC1 as a self-molecule and thus are tolerant to MUC1. Additionally, KCM mice develop spontaneous tumors of the pancreas, with tumor cells expressing large amounts of hypo-glycosylated MUC1 as observed in human PDAs (29). Therefore, the tumor growth reduction along with a significantly higher survival observed here support the potential of these therapeutic approaches in humans.

Clinically, combination treatments such as rosiglitazone and gemcitabine, FOLFIRINOX (5-FU, leucovorin, irinotecan, and oxaliplatin), monoclonal antibody and 5-fluorouracil, adriamycin, and mitomycin chemotherapy, or gemcitabine and nab-paclitaxel have been shown to significantly reduce tumor progression and metastases and significantly extend overall patient survival (1, 6–8). While those treatments led to some improvements and extended overall survival in small subsets of patients (8, 9), improved approaches to treat patients with pancreatic cancer are required (3, 10).

Clinical trials with combinations of antibodies to specific tumor antigens along with IL-2 treatment have not shown significant efficacy (22, 73). Modified IL-2 formulations alone led to longer IL-2 half-life, but without significant clinical benefit when used as a monotherapy (74). We have previously demonstrated that treatment with an anti-tumor antigen antibody and a fusion protein bestowing prolonged IL-2 signaling (i.e., Lip-MSA-IL-2 used here) led to significantly improved survival in a melanoma mouse model (22). Furthermore, in a murine model, the sustained persistence of IL-2 signaling enhanced the antitumor effects of peptide vaccines (75), highlighting the key role of sustained IL-2 signaling activation in successful immunotherapy. Although PDA is classically resistant to immunotherapy and lacks baseline T cell infiltration (76), higher clinical benefits were observed when immunotherapy/chemotherapy/chemoprevention combinations were used (41, 77). Indeed, our data support the benefits of sustained IL-2 signaling when combined with the specific tumor targeting antibody TAB004, as Lip-MSA-IL-2, or TAB004 treatments alone had no effects on survival, whereas the combination was associated with clearly improved survival.

Immunological responses observed in the PDA models following treatment in part mimic those observed and summarized earlier (22) in a murine melanoma model, including critical interactions between various effectors during administration of cancer immunotherapy. In particular, the administration of anti-MUC1 antibodies leads to effective tumor cell killing by antibody-dependent cell-mediated cytotoxicity/phagocytosis (ADCC/ADCP) in part through NK cell and/or macrophage-mediated killing activities (24, 32, 42). Remarkably, populations of CD45+CD11b+ cells (including macrophages), but not NK cells, were increased in tumors treated with Lip-MSA-IL-2 and TAB004, suggesting a key role for CD45+CD11b+ cells (including macrophages) in the limitation of tumor progression *in vivo*. Our observation is supported by the required role of macrophages in the anti-MUC1 tumor response *in vivo* (42). Notably, overall survival of PDA patients who had alterations in the genes for CD45 (*PTPRC*) and CD11b (*ITGAM*) was significantly lower than the survival of all patients with PDA (CBioportal.org query, Supplemental Figure 5S) (78, 79). Future depletion studies will be required to confirm that the CD45+CD11b+ cells involved here are macrophages.

This immune response was complemented with a significant decrease in immune regulatory cells (CD8+CD69+ cells). Interestingly, enhanced anti-tumor immunity against MHC class I tumors (RMA-S ad RM-1) was reported in CD69 knockout mice and mice treated with an anti-CD69 antibody (80). Indeed, CD8+CD69+ T cells are immunoregulatory cells that are known to promote tumor progression by inducing the production of indoleamine 2,3, dioxygenase (IDO) (62). Our previous studies indicated that IDO, one of the major players in immune tolerance but also in tumor progression, metastasis, and angiogenesis, is overexpressed in MUC1-expressing PDA (44). Thus, tMUC1 expression may contribute toward a highly tolerogenic tumor microenvironment by influencing the IDO/tryptophan pathways.

Our data suggest that the increased percent of tumor infiltrating CD45+CD11b+ cells and serum MPO concentrations are associated with the increased survival observed in the mice treated with the combination. MPO is produced especially during degranulation of neutrophils and macrophages, leading to the generation of hypochlorous acid that is commonly indicative of cellular cytotoxicity. In contrast with previous increases in neutrophils associated with Lip-MSA-IL-2 treatment in the melanoma model (22), no significant changes in neutrophil populations were observed in this study. Nevertheless, we do report that tumor progression is correlated with an increase in the number of blood neutrophils. Interestingly, the blood neutrophil/lymphocyte ratio, an independent prognostic marker of tumor progression (i.e., the lower the blood neutrophil/lymphocyte ratio, the better the outcome (65, 66), was determined to be lower in mice treated with TAB004 alone or with the combination of TAB004 + Lip-MSA-IL-2. Furthermore, the ratio of neutrophils/T lymphocytes per gram of tumor (approximated using the sum of CD45+CD4+ cells and CD45+CD8+ cells) was also lower in the tumors from mice treated with the combination of TAB004 + Lip-MSA-IL-2.

We also detected increases in serum IL-5 and CXCL1 concentrations and decreases in serum IL-6 concentrations in mice treated with the combination vs. control mice. In particular, the significant increase in circulating CXCL1, along with the correlation of the number of blood neutrophils with the tumor size, may be related to the recruitment of tumor entrained neutrophils (TENs) from the bone marrow into possibly other organs. TENs are associated with inhibiting seeding in the metastatic niche (81) by generating H_2_O_2_ and tumor secreted MCP1 (also noted in our treatment group) which are both critical mediators of anti-metastatic entrainment of stimulated neutrophils. IL-6 is a critical pleiotropic cytokine associated with innate immunity and cancer; it is known to inhibit expression of CXCL1, and is a prominent target for clinical intervention (82). Together, these data hint that the combination treatment may be associated with wound healing and macrophage/monocyte recruitment.

The presence of plasma IgG antibodies specific to tMUC1 has been associated with survival benefits in patients with breast, lung, pancreatic, ovarian and gastric carcinomas (24). Interestingly, circulating shed tMUC1 accurately detected tumor stage progression in PDA patients (27). Possible mechanisms by which anti-tMUC1 antibodies prevent tumor progression include enhanced NK cell anti-tumor activity (42), restoration of cell-cell interactions altered by tumor-associated MUC1 (24), and prevention of tMUC1-associated reduction of T cell proliferation and anergy of cytotoxic T cells (23, 31). Interestingly, the inhibition of human T cell responses by cancer-associated MUC1 was abrogated by IL-2 (31). Moreover, when conjugated to tMUC1 antibody, IL-2 stimulated the proliferation of activated human lymphocytes *in vitro* and triggered resting NK cells to lyse tumor cells (23). Furthermore, the IL-2-antibody complex promoted antitumor immunity in mice by activating tumor-reactive CD8+ T cells (20). Previous imaging analyses clearly indicated strong co-localization of TAB004 and tumor cells (43, 57), and our data highlight clear tumor responses to combined TAB004 + Lip-MSA-IL-2 immunotherapy. The neutralization of tMUC1 in circulation is likely due to TAB004 complexing with circulating tMUC1, which in turn dampens the tMUC1-induced immune suppression. This enables immune effector cells (in this case, the macrophages) to elicit an anti-tumor immune response and enhance survival. Although TAB004 alone did not improve survival, since Lip-MSA-IL-2 has been shown to activate macrophage cytotoxicity against cancer cells (83), it is possible that Lip-MSA-IL-2 likely enhances the recruitment and activation of macrophages once TAB004 is bound to tMUC1-expressing tumor cells.

Taken together, our data, for the first time, indicate that treatment with Lip-MSA-IL-2 + TAB004 significantly improved survival in an orthotopic model, and resulted in retardation of tumor progression in a spontaneous model of PDA. Remarkably, these results are the first to demonstrate improved PDA outcomes in immunocompetent mouse models. In contrast, the use of Lip-MSA-IL-2 alone or TAB004 alone were not associated with any significant improvement in tumor burden or survival in the *in vivo* PDA models tested. Beside the benefits of TAB004 as an early monitoring approach to detect cancers earlier and monitor their progression, these data indicate that TAB004 may also have clear therapeutic benefits when combined with IL-2 to stimulate a targeted immune response. Success in developing FDA-approved TAB004-based treatments of patients with non-resectable PDA would have enormous long-term clinical impact. Furthermore, TAB004 antibody therapy may usher a new area of immunotherapy for other malignancies.

## Ethics Statement

All animal experiments were conducted following protocols approved by the Institutional Animal Care and Use Committee (IACUC) of the University of North Carolina at Charlotte. All experiments were conducted following the Guide for the Care and Use of Laboratory Animals guidelines under the supervision of Dr. Chandra Williams DVM, in an AAALAC accredited facility.

## Disclosure

PM, RP, and MW are Oncotab Inc. employees. Oncotab Inc. had no involvement in data analyses, data presentation and manuscript writing.

## Author Contributions

DD conceived, performed, and analyzed the experiments and wrote and reviewed the manuscript. LM, MW, LDR, LD, and TP performed and analyzed the experiments and reviewed the manuscript. RP, NM, and KW participated in the analysis the data, and reviewed the manuscript. PM participated in conception of the experiments and the analysis the data and reviewed the manuscript.

### Conflict of Interest Statement

The authors declare that the research was conducted in the absence of any commercial or financial relationships that could be construed as a potential conflict of interest.

## Abbreviations

ADCC: Antibody Dependent Cell Cytotoxicity
ADCP: Antibody Dependent Cell Phagocytosis
APC: Allophycocyanin
CBC: Cell Blood Counts
EGFR: Epidermal Growth Factor Receptor
FITC: Fluorescein Isothiocyanate
IFN: Interferon
IL: Interleukin
MPO: Myeloperoxidase
PDA: Pancreatic Ductal Adenocarcinoma
PE: Phycoerythrin
RBC: Red Blood Cells
TAB004: Anti-tMuc1 antibody
TIL: Tumor Infiltrating Lymphocytes
TNF: Tumor Necrosis Factor
WBC: White Blood Cells.
