# Supplementary material for: Combining the Specific Anti-MUC1 Antibody TAB004 and Lip-MSA-IL-2 Limits Pancreatic Cancer Progression in Immune Competent Murine Models of Pancreatic Ductal Adenocarcinoma
Source: Front Oncol. 2019 Apr 30;9:330. doi: 10.3389/fonc.2019.00330 (PMC6503151; doi:10.3389/fonc.2019.00330)
Supplement: Supplementary file 1 [file Data_Sheet_1.PDF]

Supplemental Material

Suppl. Table 1S: Blood cytokine concentrations (average ±SEM in pg/ml, n=3 animals per treatment group)

	Treatment			
Cytokine	PBS	IL-2	TAB004	TAB004+IL-2
IL-2	9.2±1.2	18.5±1.8	10.4±1.8	22.8±4.9
IFNγ	16.1±0.8	17.7±3.2	14.4±0.7	17.1±3.8
GM-CSF	23.5±1.1	20.8±0.4	23.8±1.7	24.7±1.3
IL-12	58.2±13.6	41.0±14.9	84.4±11.1	51.1±50.4
TNFα	137.5±15.2	107.6±0.6	115.7±11.4	135.0±28.0
RANTES	125.1±90.0	544.6±469.7	112.5±25.8	91.9±15.7
IL-4	5.8±2.7	6.9±0.1	5.3±1.2	9.0±4.4
IL-5	6.0±2.3	84.0±65.2	7.8±1.7	299.9±266.2
IL-10	245.8±0.1	259.6±33.6	232.0±38.7	251.8±77.8
IL-13	607.3±23.5	601.2±50.7	766.7±231.7	610.8±259.8
IL-6	1177.0±749.1	171.9±44.1	1279.0±633.6	343.5±281.6
IL-9	515.3±317.7	721.5±229.5	1038.0±290.1	962.5±487.3
MCP1	76.2±6.5	95.4±32.2	78.4±4.0	86.8±20.9
IL1α	26.6±8.6	44.1±7.1	45.1±12.6	57.9±18.4
MCSF	54.9±7.3	7.0±2.2	22.7±15.9	62.9±1.0
VEGF	50.3±27.6	47.0±18.6	116.9±53.1	77.0±55.7
CXCL1	71.1±0.5	103.7±69.3	129.6±86.4	295.8±171.9

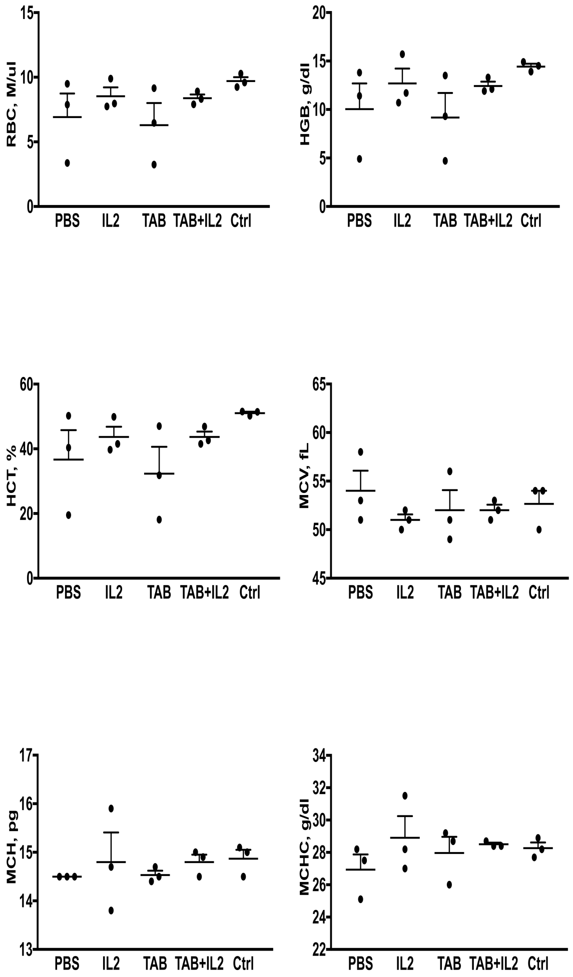

Suppl. Fig 1S. *Red blood cells (RBC) related measurements; numbers of RBC, hemoglobin (HGB), hematocrit (HCT), mean corpuscular volume (MCV), and mean corpuscular hemoglobin (MCH) concentration were determined. No difference was observed, regardless of the treatment or compared to control naïve animals (Ctrl; p=n.s.) (n=3 animals per treatment group). Data are presented as mean ± SEM. [Abbreviations: TAB=TAB004; IL2=Lip-MSA-IL-2].*

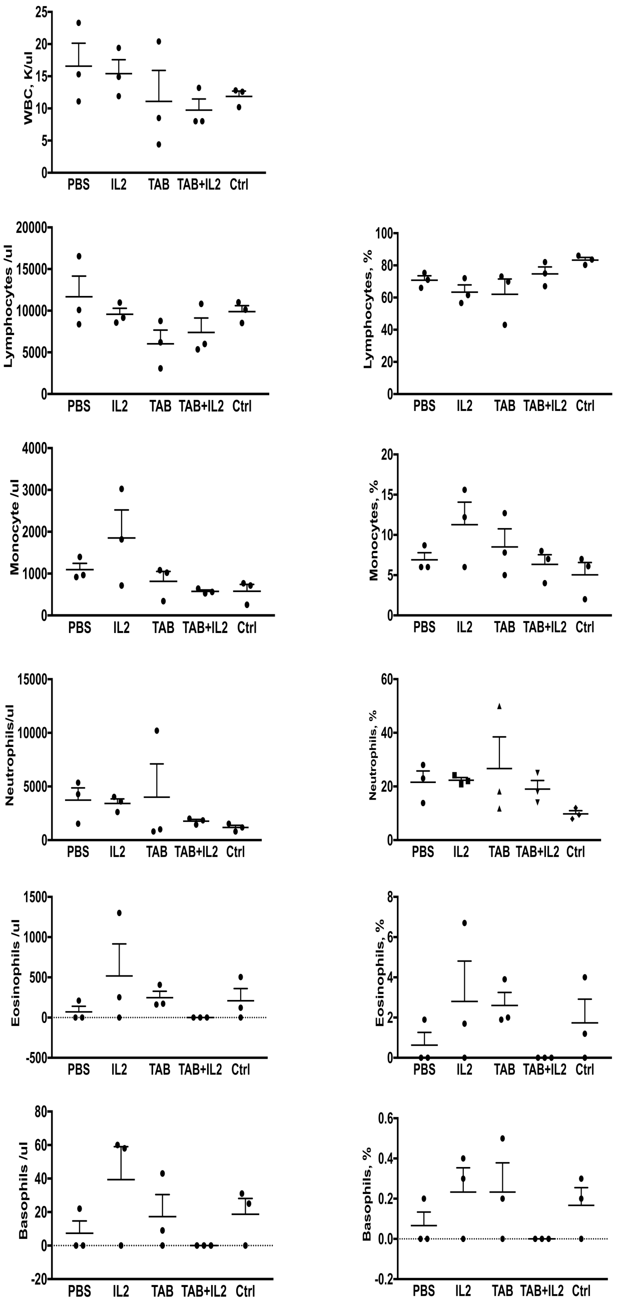

***Suppl. Fig 2S**: White blood cells (% and concentrations) and WBC subsets were numerated including lymphocytes, monocytes, neutrophils, eosinophils and basophils. WBC and WBC subsets were not different regardless of treatment and were within range of control naïve animals (Ctrl; p=n.s.) (n=3 animals per treatment group). Data are presented as mean ± SEM. [Abbreviations: TAB=TAB004; IL2=Lip-MSA-IL-2].*

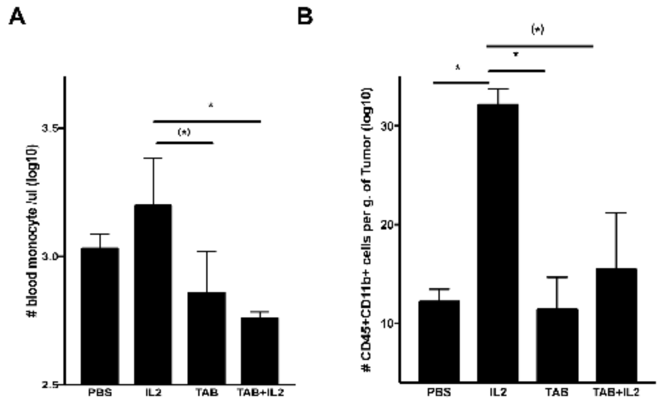

Suppl. Fig 3S*: Number of (****A****) monocyte per ul (log10) of blood and of (****B****) CD45+CD11b+ cells per gram (log10) of tumor. Monocytes were numerated and CD45+CD11b+ cells were derived from total numbers of CD45+ cells determined by flow-cytometry and normalized to one gram of tumor. Higher numbers of monocytes and of CD45+CD11b+ cells were detected in the blood and tumors, respectively, of mice treated with Lip-MSA-IL-2 alone in particular compared to mice treated with the combination TAB004+Lip-MSA-IL-2 (n=3 animals per treatment group). Data are presented as mean ± SEM. [Abbreviations: TAB=TAB004; IL2=Lip-MSA-IL-2].*

$^{(*)}p<0.08$; $^{*}p<0.05$.

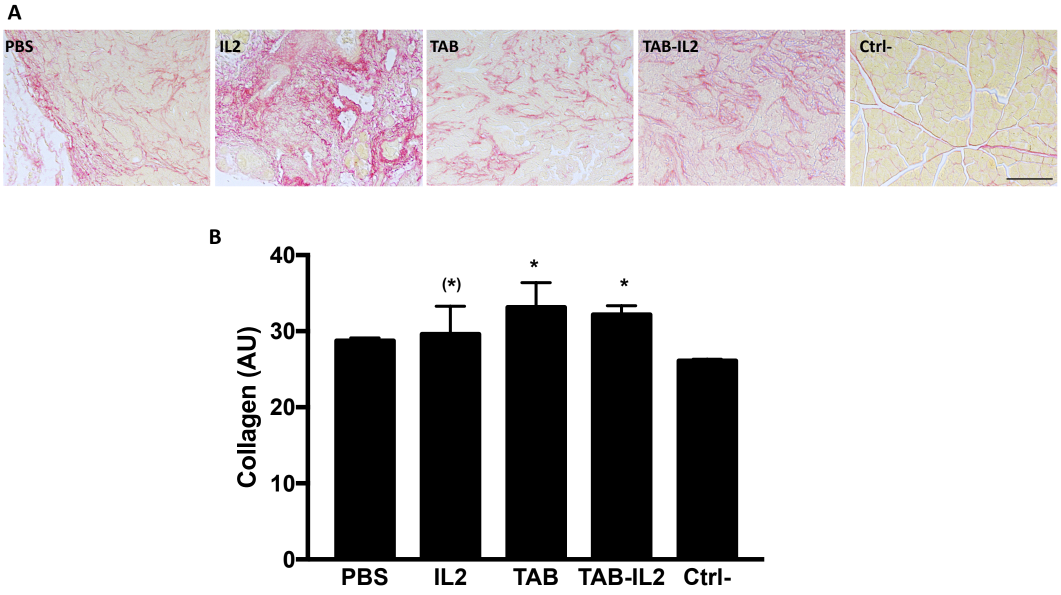

***Suppl. Fig 4S**: (**A**) Collagen accumulation in the pancreatic tumor mass on mice treated with either PBS, IL-2, TAB004 or the combination TAB004-IL-2. A control pancreas (same age, no tumor; Ctrl-) was stained along with the tumor samples using picosirius red as performed previously [70]. Furthermore, (**B**) the intensity of the collagen stain was determined using Image J. (n=3 animals per treatment group). Data are presented as mean ± SEM. [Abbreviations: TAB=TAB004; IL2=Lip-MSA-IL-2].* $^{(*)}$p<0.09; *p<0.05.

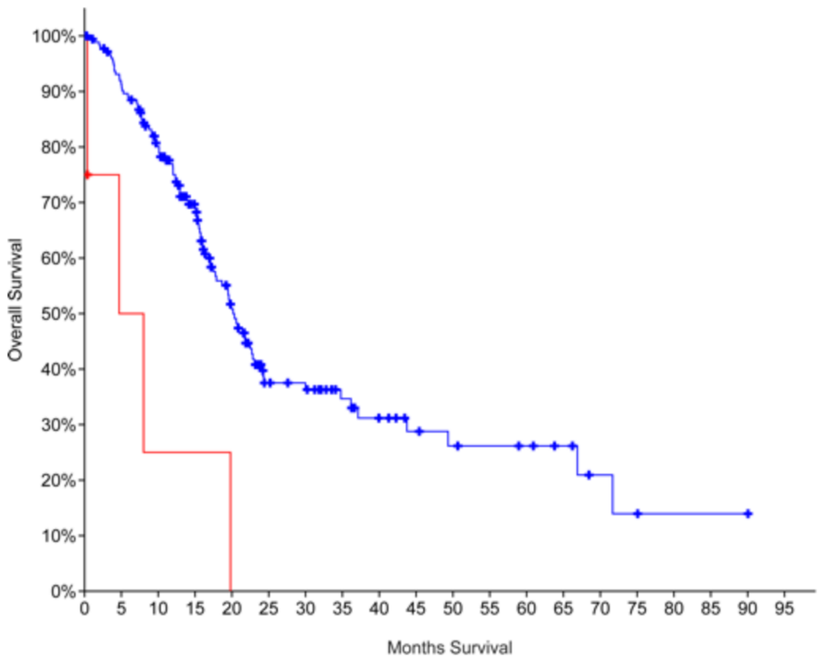

	Total #	deceased #	Survival (median, mo.)
# w/ gene alterations	4	4	4.7
# w/out gene alterations	181	96	20.2

Suppl. Fig 5S*: Overall survival of all patients with PDA (Blue) compared to overall survival of patients with PDA and mutations in CD45 (PTPRC) and CD11b (ITGAM) genes (Red) as determined by a query through CBioportal.org of the available pancreatic ductal adenocarcinoma cases (n=1033 patients; 5 studies; log-rank p=0.00173) [79; 80].*
